# Detection of immunogenic protein components in excretion/secretion products of *Acanthamoeba T5* using polyclonal antibodies

**DOI:** 10.1590/0074-02760240190

**Published:** 2025-06-09

**Authors:** Lissette Retana-Moreira, Elizabeth Abrahams-Sandí, Marco Ruiz-Campos, Johan Alvarado-Ocampo, Julián Castro, Jacob Lorenzo-Morales, Giovanni Sáenz-Arce, Antonio Osuna

**Affiliations:** 1 Universidad de Costa Rica, Facultad de Microbiología, Departamento de Parasitología, San José, Costa Rica; 2 Universidad de Costa Rica, Centro de Investigación en Enfermedades Tropicales, San José, Costa Rica; 3 Universidad Nacional, Facultad de Ciencias Exactas y Naturales, Departamento de Física, Heredia, Costa Rica; 4 Universidad de La Laguna, Instituto Universitario de Enfermedades Tropicales y Salud Pública de Canarias, La Laguna, Tenerife, Islas Canarias, Spain; 5 Universidad de La Laguna, Departamento de Obstetricia y Ginecología, Pediatría, Medicina Preventiva y Salud Pública, Toxicología, Medicina Legal y Forense y Parasitología, La Laguna, Tenerife, Islas Canarias, Spain; 6 CIBER de Enfermedades Infecciosas, Instituto de Salud Carños III, Madrid, Spain; 7 Universidad de Granada, Instituto de Biotecnología, Departamento de Parasitología, Grupo de Bioquímica y Parasitología Molecular, Granada, Spain

**Keywords:** *Acanthamoeba* T5, pathogenic potential, trophozoites, conditioned medium, extracellular vesicles

## Abstract

**BACKGROUND:**

*Acanthamoeba* is a free-living amoeba widely distributed, responsible for keratitis and granulomatous amoebic encephalitis. The presence of virulence factors in its excretion/secretion products has been demonstrated. Characterisation of these products, including the determination of immunogenic protein components using polyclonal antibodies, could be the basis for the development of new diagnostic tools and help to understand aspects related to its pathogenesis.

**OBJECTIVES:**

To identify immunogenic protein components in *Acanthamoeba* conditioned medium (ACM) and extracellular vesicles (EVs) using polyclonal anti-*Acanthamoeba* antibodies produced in the laboratory and to evaluate the effect of these antibodies in adhesion and cytopathic effect.

**METHODS:**

Excretion/secretion products were obtained after the axenic culture of a potentially pathogenic environmental *Acanthamoeba* T5 isolate. The presence of immunogenic components in lysates of trophozoites, ACM and EVs was determined using polyclonal anti-*Acanthamoeba* antibodies produced in Wistar rats. Proteomic analyses to identify the immunogenic protein components in ACM and EVs were included. Experiments to evaluate the effect of polyclonal anti-*Acanthamoeba* antibodies in adhesion and cytopathic effect *in vitro* were also performed in Vero cells.

**FINDINGS:**

Protein recognition by anti-*Acanthamoeba* antibodies in lysates, ACM and EVs was demonstrated, and these components were identified using proteomics. Decreases in adhesion and cytopathic effect after the preincubation of trophozoites with antibodies, prior to the contact with cells, were observed.

**MAIN CONCLUSION:**

The development of polyclonal antibodies, capable of recognising proteins related to pathogenesis in ACM and EVs, and with significant effects in adhesion, provides an important tool for the search for new therapeutic and diagnostic targets in infections caused by *Acanthamoeba*.

*Acanthamoeba* is a genus of free-living amoeba (FLA) widely distributed in nature and includes pathogenic isolates that can produce keratitis, encephalitis and cases of pulmonary and cutaneous manifestations. Of the 23 genotypes described to date,^1^ based on molecular analysis of the 18S rDNA gene, T4 and T5 are the most frequently isolated from nature,^2^ being T4 the responsible of most clinical cases and the most severe infections. However, there are also reports of *Acanthamoeba* keratitis and granulomatous amoebic encephalitis caused by other genotypes like T2, T5, T10 and T18,^1^ among others.

The pathogenesis during infections with *Acanthamoeba* is usually related to the presence of direct and indirect virulence factors that, together, can explain the various clinical presentations that an infected individual could develop. Osmotolerance and thermotolerance (indirect), and the secretion of proteases and cytopathic effects *in vitro* (direct) are considered the main factors related to pathogenicity.^3^ In this sense, *Acanthamoeba* can secrete several factors, independently of host cell contact, to ensure survival and adaptation within the host, like superoxide dismutases,^4^ and hydrolases, which facilitate nutrient acquisition and host damage and include glycosidases,^5^ phospholipases,^6^ ^,^ ^7^ and cysteine, metallo and serine proteases.^8^ ^,^ ^9^ ^,^ ^10^ ^,^ ^11^ However, once *Acanthamoeba* adheres to the target cells, intracellular signal transductions are also quickly activated and triggers a series of cascade effects that include phagocytosis, the secretion of proteases and the induction of apoptosis, which will eventually provoke a direct pathological damage to the host.^3^

Regardless of the mechanism of damage, most of the virulence factors of *Acanthamoeba* are transported to the extracellular milieu by classical and non-classical secretory pathways,^12^ the latter also involving the production of extracellular vesicles (EVs). Proteomic analyses of excretion/secretion products have determined the presence of free proteins related to adhesion, as well as serine and cysteine proteases, related to the degradation of extracellular matrix. Regarding EVs, different proteomic analyses by *Acanthamoeba* T4 are available,^10^ ^,^ ^13^ and some immunomodulatory properties of EVs released by different strains of genotypes T1, T2, T4 and T11 are reported.^14^ Besides, proteolytic activity of these vesicles has also been demonstrated,^15^ ^,^ ^16^ which confirms the presence of proteases as part of the cargo of EVs secreted by trophozoites of this genus.

In previous work,^15^ our research group confirmed the presence of EVs in excretion/secretion products of a potentially pathogenic environmental *Acanthamoeba* T5 isolate, incubated at two different temperatures (28ºC and 37ºC), as well as the presence of serine and cysteine proteases in conditioned media. Due to these previous results, the aim of this work was to delve deeper into excretion/secretion products of this *Acanthamoeba* T5 isolate, in order to determine the presence of immunogenic protein components using two different types of polyclonal anti-*Acanthamoeba* T5 antibodies produced in our laboratory. Moreover, we evaluated the effect of these antibodies in adhesion and cell damage produced by the amoeba to Vero cells using an *in vitro* model.

## MATERIALS AND METHODS

*Axenic culture of Acanthamoeba T5* - *Acanthamoeba* T5 (*A. lenticulata*) was isolated from a water sample of a hospital and axenically grown in 75 cm^2^ cell culture flasks with 5 mL of peptone/yeast extract/glucose (PYG) (0.75% proteose peptone, 0.75% yeast extract and 1.5% glucose) medium. Amoebae were cultured and maintained as previously reported by Castro-Artavia et al.,^17^ and molecular identification and genotyping was performed as previously described.^15^

*Preparation of excretion/secretion products of Acanthamoeba T5: conditioned medium (ACM) and isolation of EVs* - ACM was prepared by incubating culture flasks with 4 x 10^6^ trophozoites of *Acanthamoeba* for 5 h. After this time, the supernatants were collected and centrifuged at 3,500 rpm for 15 min at 4ºC to obtain an amoebae-free supernatant.

For the isolation and purification of EVs, a protocol previously described was employed,^15^ with minor modifications. For this purpose, culture flasks with 4 x 10^6^ trophozoites of *Acanthamoeba* were incubated for 5 h, at 28ºC and 37ºC, in PYG culture medium, and submitted to the same centrifugation step described for the ACM collection. The obtained supernatants were then collected and centrifuged at 17,000 xg for 30 min. After this time, the supernatants were filtered through 0.22 μm pore filters (Sartorius Minisart, USA) and ultracentrifuged at 120,000 xg in a Sorvall™ WX80 Ultracentrifuge (Thermo Fisher Scientific, USA) for 2.5 h to obtain the pellets with EVs. The pellets were washed two times in sterile phosphate buffered saline (PBS) by ultracentrifugation, resuspended in 100 µL PBS and stored at -80ºC (proteomic analyses). Characterisation analyses of EVs also included analyses of protein profiles, dynamic light scattering and atomic force microscopy, following the methodologies previously reported by our group,^15^ ^,^ ^18^ and considering the guidelines established MISEV 2023.^19^ The protein concentration of EVs was quantified using the Micro-BCA protein assay kit (Thermo Fisher Scientific, USA) and viability of trophozoites after EVs shedding was evaluated using the trypan blue exclusion test.

**Polyclonal anti-*Acanthamoeba* T5 antibodies production**

*Preparation of the antigen and immunisation* - Two different polyclonal anti-*Acanthamoeba* T5 antibodies were produced after the immunisation of four-week-old female Wistar rats with: (1) 40 μg of whole protein extract of lysates of trophozoites of *Acanthamoeba* T5 obtained as previously reported Retana-Moreira et al.^18^ or (2) 40 μg of proteins, eluted from a nitrocellulose membrane in which trophozoites of *Acanthamoeba* T5 were placed and incubated for 72 h at room temperature (with periodic addition of culture media), for further trophozoite removal and elution of proteins from the membrane (mainly secreted and surface proteins). The suspension was prepared by emulsification in complete Freund’s adjuvant (Sigma-Aldrich, USA), using a 1:1 ratio (final volume: 500 µL), and administered intraperitoneally to the rats; subsequent immunisations were performed using incomplete Freund’s adjuvant (Sigma-Aldrich, USA), for a total of 8 immunisations (1 per week).

The antibody production was evaluated using enzyme-linked immunosorbent assay (ELISA) and Western blot, as described elsewhere,^20^ employing complete lysates of different *Acanthamoeba* isolates.

*Electrophoretic separation of proteins using sodium dodecyl sulphate polyacrylamide gel electrophoresis (SDS-PAGE)* - Samples of whole protein extracts (WPE) of lysates of trophozoites were diluted 1:1 in sample buffer^21^ and subsequently loaded onto 12% SDS-polyacrylamide gels, as described elsewhere.^18^ Electrophoretic runs were performed for 90 min (120 V) and visualisation of protein bands was achieved after silver staining, following previously described protocols.^22^

The recognition of proteins in the complete extract of trophozoites of *Acanthamoeba* by polyclonal anti-*Acanthamoeba* antibodies was analysed using Western blot, as previously described by Retana-Moreira et al.^18^ Briefly, SDS-PAGE was performed and the separated proteins in the polyacrylamide gels were transferred to polyvinylidene difluoride (PVDF) membranes (Trans-Blot Turbo Midi PVDF Transfer Packs, Bio-Rad Laboratories, USA) for 40 min at 40 V in a Trans-Blot Turbo Transfer System (Bio-Rad Laboratories, USA). The membranes were subsequently blocked overnight with 5% non-fat milk in PBS - 0.1% Tween 20, washed four times in a solution of PBS-0.1% Tween 20 and incubated overnight at 4ºC with the polyclonal anti-*Acanthamoeba* T5 antibodies (dilutions 1: 3,000 for the antibody produced using the complete lysate and 1: 1,000 for the antibody produced using the eluted proteins). After the incubation, the membranes were washed and incubated for 1 h with peroxidase-conjugated goat anti-rat IgGs (1: 10,000) (Thermo Scientific, USA), and four washing steps with PBS-0.1% Tween 20 were performed prior to visualisation in a ChemiDoc Imaging system (BioRad, USA) using the Clarity ECL Western substrate (BioRad, USA).

To evaluate the specificity of the antibodies produced, lysates of Vero cells, trypomastigotes and epimastigotes of the protozoan parasite *Trypanosoma cruzi* (*T. cruzi*) and trophozoites of the FLA *Naegleria fowleri* (*N. fowleri*) were also included in the analyses.

PVDF membranes with the samples of lysates were also washed using a solution of 0.01 M Tris and 0.15 M NaCl (pH 2.3) for 24 h, followed by three washing steps with PBS-0.1% Tween 20. Then, the membranes were incubated 10 mg/mL sodium *meta-*periodate (Thermo Fisher Scientific, USA) in 0.05 M sodium acetate buffer^23^ for 4 h, at room temperature, in the dark, to eliminate carbohydrate domains in proteins through the oxidation of sugar residues and evaluate the antibody recognition of those proteins. After this treatment, the membranes were blocked, and Western blots were performed as described above.

*Western blot* - The recognition of proteins in ACM and EVs by polyclonal anti *Acanthamoeba* antibodies was also analysed using Western blot, as previously described by Retana-Moreira et al.^18^ Briefly, SDS-PAGE was performed and the separated proteins in the polyacrylamide gels were transferred to PVDF membranes (Trans-Blot Turbo Midi PVDF Transfer Packs, BioRad Laboratories, USA) for 40 min at 40 V in a Trans-Blot Turbo Transfer System (Bio-Rad Laboratories, USA). The membranes were subsequently blocked overnight with 5% non-fat milk in PBS - 0.1% Tween 20, washed four times in a solution of PBS-0.1% Tween 20 and incubated overnight at 4ºC with the polyclonal anti-*Acanthamoeba* T5 antibodies (dilutions 1: 3,000 for the antibody produced using the complete lysate and 1: 1,000 for the antibody produced using the eluted proteins). After the incubation, the membranes were washed and incubated for 1 h with peroxidase-conjugated goat anti-rat IgGs (1: 10,000) (Thermo Scientific, USA), and four washing steps with PBS-0.1% Tween 20 were performed prior to visualisation in a ChemiDoc Imaging system (BioRad, USA) using the Clarity ECL Western substrate (BioRad, USA).

*Proteome analyses of immunogenic components in EVs and ACM of Acanthamoeba T5* - Proteomic analyses of immunogenic components of ACM and EVs were performed using a bottom-up shotgun strategy, as previously described.^24^ For ACM, immunogenic protein bands recognised by anti-*Acanthamoeba* antibodies in Western blots were matched to the corresponding bands on Coomassie blue stained gels, manually excised and submitted to tryptic digestion followed by nano-LC-MS/MS for protein identification, as previously performed.^18^ Regarding EVs, due to the recognition of multiple bands in the same lane, the whole proteomic cargo was analysed. To this end, samples were mixed with an electrophoresis sample buffer and loaded into wells of 12% SDS-PAGE gels. The electrophoretic run was stopped as soon as the migration front entered three mm into the resolving gel to concentrate the whole protein cargo as a single band in the stacking/resolving gel interface, as previously described.^18^ The resulting bands were visualised by Coomassie stain, excised, destained in 50% acetonitrile, and finally submitted to tryptic digestion and nano-LC-MS/MS analysis.

As previously performed for EVs of *N. fowleri*,^18^ proteins in gel plugs were reduced with 10 mM dithiothreitol, alkylated with 50 mM iodoacetamide and digested overnight in an automated workstation (Intavis, Germany) using sequencing-grade trypsin (Sigma Aldrich, USA). The resulting peptides were dried, redissolved in water containing 0.1% formic acid and transferred to polypropylene nano-LC vials. Ten µL of each digest was loaded on a C18 trap column (PepMap^®^, Thermo Fisher Scientific, USA), washed with 0.1% formic acid (solution A), and then separated at 200 nL/min with a three µm particle, 15 cm x 75 µm C18 Easy-spray^®^ analytical column using a nano-Easy^®^ 1200 chromatograph (Thermo Fisher Scientific, USA). A gradient from 0.1% formic acid (solution A) to 80% acetonitrile with 0.1% formic acid (solution B) was developed in-line with a Q-Exactive Plus™ mass spectrometer (Thermo Fisher Scientific, USA), as previously described, for a total time of 35 min and MS spectra were acquired in positive mode at 1.9 kV, with a capillary temperature of 200ºC, using one µscan at 400 - 1600 m/z, maximum injection time of 100 ms, AGC target of 3 x 10^6^, and orbitrap resolution of 70,000. The top 10 ions with two-five positive charges were fragmented with an AGC target of 1 x 10^5^, maximum injection time of 110 ms, resolution 17,500, loop count 10, isolation window of 1.4 m/z, and a dynamic exclusion time of 5 s. The obtained MS/MS spectra were processed for peptide matching against protein sequences contained in the UniProt database for *Acanthamoeba* using Peaks X^®^ (Bioinformatics Solutions, Canada). Cysteine carbamidomethylation was set as a fixed modification, while deamidation of asparagine or glutamine, and methionine oxidation were set as variable modifications, allowing up to two missed cleavages by trypsin. Parameters for match acceptance were set to false discovery rate (FDR) < 1%, -10 lgP protein score ≥ 20, and ≥ one unique peptide.

Annotations of gene ontology (GO) for the identified proteins were performed using the Database for Annotation, Visualisation and Integrated Discovery (DAVID).

*Inhibition of adhesion and cytopathic effect assays using polyclonal anti-Acanthamoeba antibodies* - To evaluate the effect of polyclonal anti-*Acanthamoeba* T5 antibodies in the inhibition of adhesion of trophozoites over cell cultures, the protocol described by Rodríguez et al.^25^ was employed. For this purpose, 5 x 10^5^ trophozoites were incubated with 50 µL of the different polyclonal antibodies (prepared with secreted and surface proteins) for one hour, and then added to confluent Vero cell monolayers for 10, 20 and 40 min. After each time point, non-adhered trophozoites were counted using a Neubauer chamber and percentages of non-adhered trophozoites were calculated. Moreover, the percentage of adhered trophozoites was calculated by subtracting the percentage of non-adhered trophozoites to 100%.

Inhibition of cytopathic effect assays were performed using crystal violet stain as previously described.^14^ Briefly, cell cultures (1 x 10^5^) were grown until confluence in 24-well plates (Costar Corning Cell Bind, USA) and trophozoites previously incubated with each of the polyclonal antibodies (prepared with whole protein extracts of lysates of trophozoites, and secreted and surface proteins) for 1.5 h, under gentle shaking, were added over the cell monolayers and incubated in serum-free medium. Incubation of the plates was performed for 24 h at 37ºC and the cytopathic effect was assessed macroscopically after staining the wells with crystal violet.

*Acanthamoeba* T5 trophozoites not incubated with the antibodies were employed as positive controls of adhesion and cytopathic effect.

*Ethics statement* - The experiments performed using animals were approved by the Ethical Committee of the University of Granada (235-CEEA-OH-2018) and by the authorities of the Regional Government of Andalucía (JJAA) (number 12/11/2017/162). The use of animals was performed according to the institutional guidelines (Spanish government regulations) (Real Decreto RD1201/05) and the guidelines of the European Union (European Directive 2010/63/EU).

## RESULTS

**Fig. 1:**
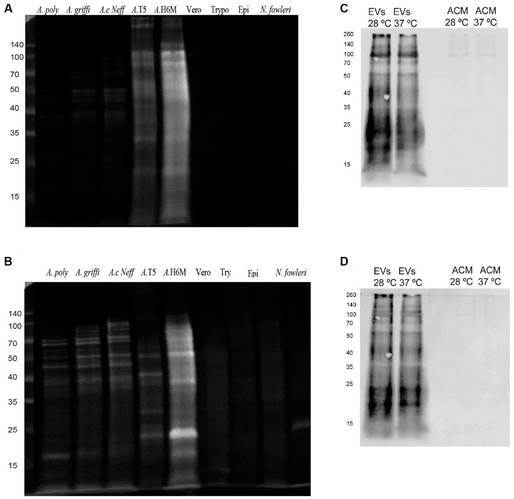
Western blot that shows the recognition of bands in protein extracts of lysates, conditioned media and extracellular vesicles of *Acanthamoeba*, by polyclonal antibodies: (A) recognition of whole protein extracts of different lysates of trophozoites of *Acanthamoeba* by antibodies prepared with whole protein extracts (WPE) of lysates of *Acanthamoeba* T5; (B) recognition of whole protein extracts of lysates of trophozoites of *Acanthamoeba* T5 by antibodies prepared with the elution of secreted and surface proteins; (C) recognition of *Acanthamoeba* conditioned medium (ACM) and extracellular vesicles (EVs) of *Acanthamoeba* T5 by antibodies prepared with WPE of *Acanthamoeba* T5; (D) recognition of ACM and EVs by antibodies prepared with the elution of secreted and surface proteins. For these experiments, the antibodies were proven against lysates of *Acanthamoeba polyphaga* (4.5 µg), *A. griffini* (4.4 µg), *A. castellanii* Neff (5.1 µg), *A. lenticulata* (T5) (3.2 µg) and *Acanthamoeba* T4 isolate H6M (17.7 µg); whole protein extracts of Vero cells (17.7 µg), trypomastigotes (3.7 µg) and epimastigotes (11.2 µg) of the parasite *Trypanosoma cruzi*, and trophozoites of the free-living amoeba *Naegleria fowleri* (3.0 µg) were also included. For A and B, 12 µL of each sample was loaded onto the gel while, for C and D, 9 µg of each sample were loaded. The same membrane with proteins was employed for both incubations with the antibodies (exhaustive washing steps to remove the previous antibodies and confirmation of the washing steps were performed).

*Preparation of excretion/secretion products of Acanthamoeba T5: ACM and isolation of EVs* - Under our experimental conditions, protein concentrations of 7 µg/µL and 1 µg/µL were obtained for ACM and EVs, respectively. EVs isolated from trophozoites incubated at 28ºC and 37ºC revealed a similar protein profile in SDS-PAGE [Supplementary data (Fig. 1)]. Additionally, dynamic light scattering (DLS) analyses also indicated similar sizes of EVs from trophozoites incubated at 28ºC (128.7 ± 52.77 nm) and 37ºC (128.2 ± 61.54 nm). The viability of trophozoites after the incubation time was over 98%.

**Fig. 2:**
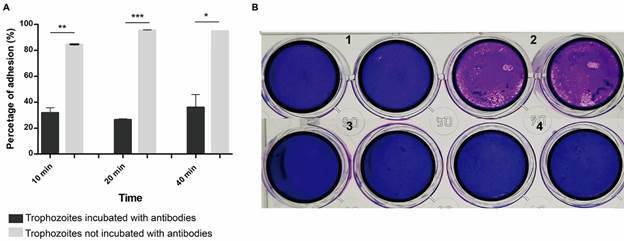
effect of polyclonal anti-*Acanthamoeba* antibodies (prepared with the elution of secreted and surface proteins) over adhesion and cytopathic effect of trophozoites of *Acanthamoeba* T5. In this figure, an effect of polyclonal anti-*Acanthamoeba* antibodies over the adhesion of trophozoites was observed, as percentages of adhesion decreased after the incubation of cells with pretreated trophozoites of the amoeba (A). Additionally, the cytopathic effect of trophozoites of *Acanthamoeba* over the Vero cell monolayer was also inhibited when employing pretreated trophozoites in the experiments (B), as no disruption of the monolayer was observed when applying the crystal violet stain: 1. control cells; 2. trophozoites of *Acanthamoeba* not pretreated with polyclonal antibodies; 3. trophozoites of *Acanthamoeba* pretreated with polyclonal antibodies (vs. secreted and surface proteins); 4. trophozoites of *Acanthamoeba* pretreated with polyclonal antibodies (vs. proteins of the whole protein extracts - WPE - of the complete lysate of trophozoites).

*Production of polyclonal anti-Acanthamoeba antibodies: analyses using Western blot* - Polyclonal anti-*Acanthamoeba* antibodies prepared by the immunisation with WPE or an elution of secreted and surface proteins recognised a wide variety of components of both high and low molecular weights, as shown in Fig. 1. While the antibodies prepared with the WPE of *Acanthamoeba* T5 provided a more specific identification (Fig. 1A), antibodies prepared with secreted and surface proteins detected a larger number of bands in extracts of all strains; however, a slight cross-reactivity with other protozoa and Vero cells was observed (Fig. 1B). The use of *meta*-periodate eliminated this cross-reactivity, as shown in Supplementary data (Fig. 2).

*Recognition of protein components in excretion/secretion products by polyclonal antibodies and proteomic analyses* - Polyclonal anti-*Acanthamoeba* antibodies were also challenged with excretion/secretion products. Results shown in Fig. 1C-D reveal multiple protein bands in EVs from both temperatures, recognised by both types of antibodies; on the contrary, a very slight recognition of ~three bands (approximately 100, 140 and 260 kDa) present in conditioned media, and more evident at 28ºC, was observed when the antibody prepared using the whole protein extract of the lysate was employed.

Due to the presence of these multiple bands, the whole proteomic cargo of EVs was analysed. Under the methodology employed, a total of 227 (28ºC) and 243 (37ºC) proteins were identified in EVs, 8.16% and 10% corresponding to uncharacterised proteins, respectively [Supplementary data (Tables I-II)]. Of particular interest resulted in the identification of serine and cysteine proteases, elongation factor-1 alpha, several types of peptidases (including aminopeptidases), glycerol-3 phosphate dehydrogenases, actin, coronin, myosin, heat shock proteins (HSP-90), among others. Venn diagram from Fig. 2 reveals that more than 85% are shared proteins in EVs secreted at both temperatures. Annotations of GO for identified proteins were also performed and included in the same figure.

Regarding ACM, two of the three bands recognised by polyclonal anti-*Acanthamoeba* antibodies using Western blot were matched to the corresponding bands on Coomassie blue stained gels. Proteomic analyses of two of these bands revealed 13 (two uncharacterised) and 30 (three uncharacterised) proteins for bands of molecular weights of approximately 140 and 260 kDa, respectively. A variety of hydrolases, peptidases and mannose-binding proteins were identified [Supplementary data (Tables III-IV)].

**Fig. 3:**
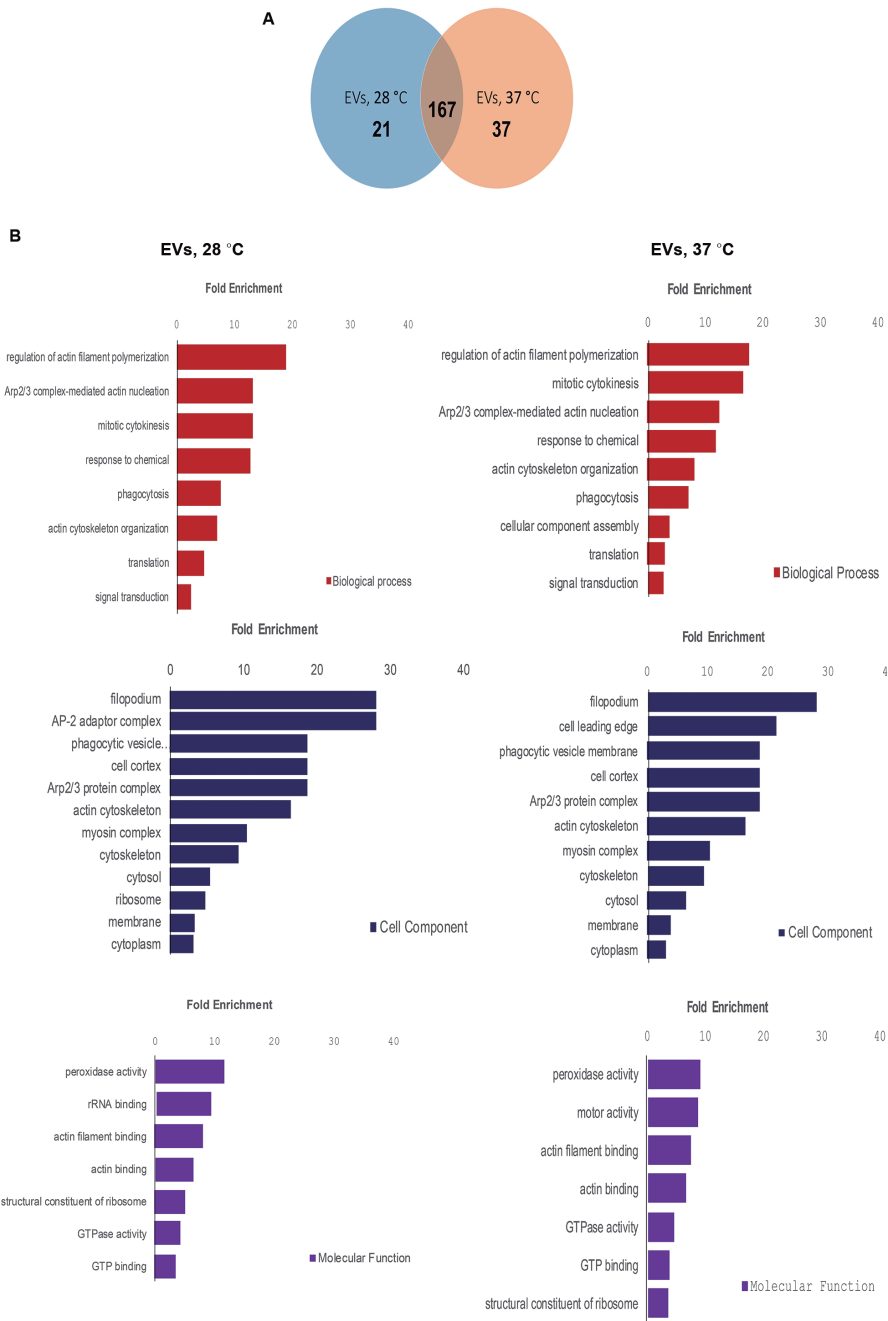
protein identification in extracellular vesicles of *Acanthamoeba* T5 secreted at two different temperatures: (A) Venn diagram of specific and shared proteins of extracellular vesicles (EVs) of *Acanthamoeba* T5 collected after the incubation of trophozoites at 28ºC and 37ºC (excluding uncharacterised proteins); (B) gene ontology (GO) of the protein cargo (biological process, cellular component and molecular function terms.

*Inhibition of adhesion and cytopathic effect of Acanthamoeba T5 trophozoites by polyclonal anti-Acanthamoeba antibodies* - The effect of polyclonal anti-*Acanthamoeba* antibodies (prepared using the elution of secreted and surface proteins) over adhesion and cytopathic effect of trophozoites of the amoeba were evaluated using an *in vitro* model with Vero cells. Results revealed that the mean percentage of adhesion of non-antibody pretreated amoeba incubated with cells for 10 min was 84.43%, while a mean percentage of 31.81% was obtained when amoebae were previously incubated with the polyclonal anti-*Acanthamoeba* antibodies (Table). At 40 min of incubation, percentages obtained were 94.81% for non-pretreated amoeba and 35.92% for amoebae previously incubated with the polyclonal anti-*Acanthamoeba* antibodies*.* For both times, results showed statistically significant differences in percentages of adhesion of amoebae over Vero cells (Fig. 3A). Regarding cell damage observed after the crystal violet stain, wells in which trophozoites of *Acanthamoeba* previously incubated with polyclonal antibodies were incubated with cells did not show the characteristic disruption of the monolayer by trophozoites, previously reported for this isolate and observed when non treated trophozoites were incubated with the cells (Fig. 3B).

**TABLE:** Mean percentage of adhesion of antibody pretreated amoebae incubated with cells vs. non antibody pretreated amoebae Antibody pretreated trophozoites

Time	10 min	20 min	40 min
Replicate 1	29	27	28.85
Replicate 2	34.62	25.96	43
Mean	31.81	26.48	35.93
SD	2.81	0.52	7.08
Non antibody pretreated trophozoites			
Replicate 1	84.24	95.39	94.81
Replicate 2	84.62	97.77	94.81
Mean	84.43	95.58	94.81
SD	0.19	0.19	0

SD: standard deviation.

## DISCUSSION

*Acanthamoeba* is a genus of free-living amoebae that includes some species capable of producing keratitis, granulomatous amoebic encephalitis and skin lesions. Of worldwide distribution, this amoeba can be isolated from a variety of environments related to human activity, being able to act as facultative parasites in immunosuppressed individuals, or in people with particular risk factors like the use of contact lenses.

Despite genotype T4 has the highest prevalence among clinical isolates from opportunistic infections from humans and animals,^26^ ^,^ ^27^ being the responsible of most clinical cases and the most severe forms of disease, infections produced by atypical *Acanthamoeba* genotypes, like genotype T5, could be associated to worse prognosis and resistance to therapy.^28^ In previous research performed by our group, it was possible to axenically grow an *Acanthamoeba* T5, isolated from a water source and identified as *A. lenticulata* by molecular approaches (accession number: MH82415).^14^

Due to the importance of the origin of the water source (a hospital), we decided to evaluate the pathogenic potential of this isolate, including techniques to determine osmo- and thermotolerance, as well as to evidence the secretion of proteases and the effect of trophozoites over cell monolayers, experiments that concluded confirming that the isolate is thermotolerant at 37ºC, and produces serine and cysteine proteases; the cytopathic effect over Madin-Darby canine kidney (MDCK) and Vero cell monolayers was also demonstrated. In the same work, the secretion of EVs by trophozoites of this T5 isolate was also confirmed, and experiments were conducted to characterise and compare EVs secreted by trophozoites that were incubated at 28ºC and 37ºC, without deepening into cargoes and functional roles of the vesicles.

Results obtained from this previous experience motivated us to delve deeper into characterising excretion/secretion products of this T5 isolate, in which protein recognition analyses using polyclonal anti-*Acanthamoeba* antibodies produced in rats were included. Moreover, the effect of these antibodies in the inhibition of adhesion and cell damage by trophozoites, using an *in vitro* model of infection, was also accessed. 

Protein recognition by polyclonal antibodies obtained after the immunisation of rats with whole protein extracts of lysates of trophozoites, and an elution of secreted and surface proteins, was demonstrated using Western blot. In this sense, several bands of molecular weights between ≥ 15 kDa to ≥ 260 kDa were recognised by the antibodies in whole protein extracts of complete lysates of different *Acanthamoeba* isolates (Fig. 1), including our *Acanthamoeba* T5 isolate. Moreover, the treatment of the membrane with sodium *meta-*periodate prior to the challenge with the same antibodies resulted in the recognition of less bands [Supplementary data (Fig. 2)], confirming the presence of non-glycosylated proteins that could be responsible, at least in part, for cross-reactions with other types of cells or microorganisms employed in this study.

Using these polyclonal antibodies, it was also possible to identify immunogenic protein components in both ACM and EVs (Fig. 1). While, for ACM, the recognition of only three protein bands of molecular weights of ≥ 100 kDa was evidenced (approximately 100, 140 and 260 kDa), several bands of a wider range of molecular weights (≥ 15 to ≥ 250 kDa) were recognised in EVs collected at each temperature. The latter results of high interest since protein recognition by the antibody produced using secreted and surface proteins could suggest the presence of proteins involved in the adhesion of the trophozoite, proteins that could also be part of the cargo of EVs. In a previous study,^14^ differences in the protease content between EVs secreted at 28ºC and 37ºC were suggested, since a higher protease activity of the EVs obtained at 37ºC was evidenced in the zymographic analyses using gelatin. In this work, proteomic analyses of EVs secreted at both temperatures were performed, revealing a very similar cargo, with only very slight differences in abundance and types, as shown in Fig. 2. GO analyses showed that the most represented GO terms in the category molecular function were “GTPase activity”, “GTP binding”, “actin binding”, “peroxidase activity”, “motor activity” and “structural constituent of ribosome” (Fig. 2). The presence of protein components in EVs that could have a role in processes like phagocytosis, endocytosis and cell motility, among others, supports the participation of EVs in amoeba-cell interactions and hence, in the pathogenic process during *Acanthamoeba* infections. In addition, differences in enzymatic profiles, metabolic processes and potential distinct origin of EVs from different strains of *Acanthamoeba* could even shed light onto the diversity and complexity of these vesicles, with direct implications for understanding host-pathogen interactions, disease mechanism and developing new therapies for the clinical intervention of *Acanthamoeba*-related diseases.^12^ Overall, protein types in the excretion/secretion products (including EVs) encompassed a wide variety of proteases and peptidases, heat shock proteins, mannose-binding proteins, GTPases, and proteins related to endocytosis and cell motility.

Regarding the secretion of proteases, it is well known that these are involved in plasminogen activation and collagen and fibronectin degradation,^29^ as well as in tissue invasion, not by the direct process of cellular lysis, but by digestion of the extracellular matrix.^9^ Since in *Acanthamoeba* proteases act upon plasminogen, collagen and fibronectin, these are considered necessary for invasion, degradation of connective tissue and alteration of cell permeabilisation.^30^ ^,^ ^31^ ^,^ ^32^ ^,^ ^33^ ^,^ ^34^ For *A. castellanii*, Rico et al. suggested that proteases with weights between 50-250 kDa could be cysteine proteases capable of producing the degradation of iron-binding proteins.^32^ Previous studies have also demonstrated the presence of aminopeptidases in the secretion products of *Acanthamoeba,* ^33^ ^,^ ^34^ ^,^ ^35^ suggesting that these enzymes could affect cell adhesion and favours phagocytosis through disruption by the amoeba.

Polyclonal anti-*Acanthamoeba* antibodies produced in our laboratory were also employed to evaluate their role in the inhibition of adhesion and cytopathic effects. For these analyses, *Acanthamoeba* trophozoites were pretreated with the antibodies prior to the incubation with Vero cells, and the effect of these antibodies was analysed after counting trophozoites that remained in the supernatant (adhesion assays), or after crystal violet stain of monolayers incubated with these trophozoites (cytopathic effect assays). In these experiments, results revealed significant differences in percentages of adhesion of amoebae over Vero cells when using antibody-pretreated trophozoites, when compared to the use of non-pretreated trophozoites. As described before, it is known that once *Acanthamoeba* adheres to the target cells, intracellular signal transduction is quickly activated and triggers a series of cascade effects that will eventually provoke a direct pathological damage to the host.^3^ Therefore, this result confirms the possibility of employing these type of antibodies to prevent adhesion of the amoeba to the cells, and the consequent events this adhesion process brings.

For the evaluation of the role of antibodies over the cytopathic effect, wells in which antibody-pretreated trophozoites of *Acanthamoeba* were incubated with cells did not show the characteristic disruption of the monolayer observed when non-pretreated amoebae were employed (Fig. 3); results were the same regardless of the type of antibody used. These results coincide with a previous report using trophozoites of *Acanthamoeba castellanii,* in which a decrease of 90% in adhesion of trophozoites to fibroblasts was reported by Rodríguez et al.^25^ In 2005, Garate et al. have also demonstrated that polyclonal anti-mannose-binding protein antibodies, prepared in chickens, were potent inhibitors of *Acanthamoeba* adhesion to epithelial cells, after evidencing the key role of this protein in the pathogenesis of *Acanthamoeba* infection by mediating host-parasite interactions.^36^

Research related to this environmental *Acanthamoeba* T5 isolate is still ongoing, since characterisation analyses revealed interesting results. The development of polyclonal antibodies that are capable of recognising proteins related to pathogenesis in EVs, that also have an important effect in adhesion, provides an important tool for the search for new therapeutic and diagnostic targets in infections caused by *Acanthamoeba*. More studies, including a wider variety of techniques, are necessary to elucidate the specific roles of EVs of *Acanthamoeba* in pathogenesis; in this sense, multi-omics techniques, such as the metabolite, protein, and lipid extraction (MPLEx) coupled to mass spectrometry^37^ and RNA-sequencing,^38^ could be employed to comprehensively understand how these vesicles might be involved in the regulation of gene expression, metabolism, virulence and microbial adaptation, among others, from a more dynamic and interactive perspective,^24^ ^,^ ^39^ ^,^ ^40^ ^,^ ^41^ as suggested by Medeiros et al.^12^
