# Supplementary material for: Detection of immunogenic protein components in excretion/secretion products of Acanthamoeba T5 using polyclonal antibodies
Source: Mem Inst Oswaldo Cruz. 2025 Jun 9;120:e240190. doi: 10.1590/0074-02760240190 (PMC12158434; doi:10.1590/0074-02760240190)
Supplement: Supplementary file 1 [file 1678-8060-mioc-120-e240190-s.pdf]

TABLE I

Identified proteins in extracellular vesicles (EVs) secreted by trophozoites of *Acanthamoeba* T5 incubated at 28°C

Protein group	Accession	Protein	Mass (Da)
1	L8HCZ7	Actin-1, putative	41676
2	L8GXI3	Rho family, small GTP binding protein Rac3, putative	21686
3	O77202	Myosin-IA	134104
4	L8H9Q4	Ribosomal protein s27a, putative	17847
5	O61080	Myosin IC heavy chain	129461
6	L8H836	V-type proton ATPase proteolipid subunit	16580
7	P90630	Myosin-I binding protein Acan125	121610
8	L8HEA2	I/LWEQ domain containing protein	70416
9	L8GRX4	Uncharacterised protein	21176
10	L8HGB4	RabE family small GTPase (Fragment)	17380
11	L8HKB9	Conditioned medium factor, putative	77482
12	L8H6F1	p53 inducible protein	163453
13	L8GJT1	Linoleate 8R-lipoxygenase	134070
14	L8GI20	Uncharacterised protein (Fragment)	18530
15	L8HD31	Ras-related protein ORAB-1, putative	23048
16	L8HFB5	Glutamate dehydrogenase	51265
17	L8H373	14_3_3 domain-containing protein	36290
19	L8H4N4	Uncharacterised protein	22477
21	L8HCM5	Calponin domain containing protein	67120
22	L8H6G0	Guanylate cyclase	66526
23	L8GHW5	C8 sterol isomerase	21546
25	L8HD62	Acyloxyacyl hydrolase	66359
26	L8GW48	PAKA subfamily protein kinase	56680
27	L8H896	RAB1B, member RAS oncogene family	22332
28	L8GVM5	Talin, putative (Fragment)	120262
29	L8GUQ6	Uncharacterised protein	33579
30	L8H8P6	Ras-like protein 1, putative	22559
31	L8GD76	Terpene cyclase/mutase family member	81940
32	L8GXE8	Ras subfamily protein, putative	33152
33	L8GR43	RAP1A, member of RAS oncogene family	22280
34	H2EZP3	Catalase	55286
35	L8HHF3	Ras-related protein Rab-7A	20543
37	L8GG53	Longin domain-containing protein (Fragment)	21563
38	L8GQE7	Vacuolar protein 8	54138
39	L8GIH3	Ras family GTPase	21879
40	L8H806	Ras-related protein Rab-1A, putative	19134
41	L8GZK1	Elongation factor 1alpha, somatic form, putative (Fragment)	35896
43	L8HIM5	Acidstable alpha-amylase	53108
44	L8HGZ3	Rab8/RabEfamily small GTPase, putative	22943
45	L8GZY0	Ras-related protein Rac1, putative	22515
47	L8GZS5	Ras GTPase rap1b, putative	21717
48	L8H1F4	Myosin VIIa, putative	232883
49	L8HJ59	F-actin-capping protein subunit beta	31162
50	L8HAC7	Ras-related protein Rab	24602
51	L8GGY5	ABC2 type transporter superfamily protein	169171
52	L8H4B9	SH3 domain containing protein	40786

CC BY

Protein group	Accession	Protein	Mass (Da)
53	L8GMR7	Eukaryotic porin protein	33586
54	L8GNN8	LIM domain containing protein	18989
56	H9C880	Protein kinase C4 (Fragment)	40357
57	L8HGF1	Ras subfamily protein	21544
58	L8GUU1	Serine/threonine kinase	39217
59	L8H7M2	Myosin1, putative	177415
60	L8HJG5	Cell cycle control protein	36863
63	L8HJ94	Rac-like protein (Fragment)	24794
65	L8GLB7	Purinergic receptor, putative	39667
66	L8GSY8	Uncharacterised protein	131967
67	L8GVH2	Phospholipid-transporting ATPase	129759
69	L8H4Z4	Guanylate cyclase	176363
70	L8HG55	Ras subfamily protein	18012
71	L8GLH7	DIL domain containing protein	74709
72	L8H8C6	Protein kinase domain containing protein	52031
73	L8H850	Ras-related protein Rap-1, putative	21517
74	L8HED5	C2 domain containing protein	14171
75	L8GW76	RAC GTPase	18654
76	L8HJ50	Uncharacterised protein	22572
77	L8HHZ1	V-type proton ATPase subunit a	92623
78	H9C899	Protein kinase C23	43345
79	L8GT20	Eukaryotic translation elongation factor 2, putative	93342
80	L8GHJ9	Gtpase-activator protein for Ras family gtpase	283475
81	B0FYM3	Serine proteinase	43788
82	L8GQ45	Peptidyl-prolyl cis-trans isomerase	18101
83	Q2TTE4	HSP70 (Fragment)	52239
84	L8H4P8	Guanine nucleotide-binding protein beta subunit, putative	35215
85	L8GNH8	60S ribosomal protein L7a	31732
86	L8HGS9	Rho guanine dissociation factor isoform 2, putative	22716
87	L8HCW6	PPOD1 peroxidase	14055
88	H9C898	Protein kinase C22	45499
89	L8HM01	Uncharacterised protein	57527
90	L8GTD0	Chorismate mutase subfamily protein	20759
91	L8GF41	14_3_3 domain-containing protein	29440
92	L8H9P5	DOCK family protein	190306
93	L8H8F6	Phosphoesterase family protein	57246
94	Q6QU59	Coronin	50143
95	L8GQT3	40S ribosomal protein S3a	29244
96	L8GLU0	Ribosomal protein S4, putative (Fragment)	21839
97	L8H082	Communication mutant protein f, putative	116632
98	L8H5T5	Guanylate cyclase	168120
100	L8H1E5	F-actin-capping protein subunit alpha	30721
101	L8GWZ2	Arp2/3 complex 34 kDa subunit	33355
102	L8HDK5	Actin-related protein 3, putative	47983
103	L8H0P3	40S ribosomal protein S12	16613
104	L8GI71	S-adenosylmethionine synthase	42531
105	L8GF83	Peroxiredoxin 2, putative	22119
107	L8HCU4	Guanine nucleotide binding protein G(O), alpha subunit, putative	40681

Protein group	Accession	Protein	Mass (Da)
109	L8GUS9	Ras-like protein	27427
112	L8GUC9	Myosin head (Motor domain) domain containing protein	163768
113	L8GFZ9	Coronin	50119
114	L8H192	Actin-related protein 2/3 complex subunit 5	13950
115	L8GK33	Amine oxidase	76094
116	L8GXA8	Copine VIII, putative	64292
117	L8GM46	Uncharacterised protein	66861
118	L8HBH8	Non-specific serine/threonine protein kinase	47151
119	L8GLV9	Cell division control protein 42, putative	24461
120	L8HA95	Rho-GEF domain containing protein	96754
122	L8GG08	Actin bundling protein	31046
123	L8HE57	C2 domain containing protein	14182
124	L8H6R4	Peroxidase ppod1, putative	14178
125	L8HE67	Ankyrin repeat containing protein	16126
126	L8H6I9	SH3 domain containing protein	56923
127	L8H7I0	ABC transporter, ATP binding domain containing protein	75060
128	L8HBF6	Profilin	13084
129	L8GFL5	Peroxidase	23233
130	L8HK96	Uncharacterised protein	18951
131	L8H015	Uncharacterised protein	234320
132	L8H0Z3	Uncharacterised protein	190828
133	L8GY18	Protein kinase domain containing protein	111296
134	L8GPZ0	Laminin egf-like (Domains iii and v) domain containing protein (Fragment)	193141
135	L8GVG0	Toll-like protein	33223
136	L8H599	Ras-related protein ralB-B, putative	20317
137	B0FYM4	Encystation-mediating serine proteinase	42797
138	L8HJ43	RAB11B protein, putative	23438
139	L8HC07	ABC2 type transporter superfamily protein	158067
140	L8H122	Vacuolar proton pump d subunit, putative	35205
142	L8HKL1	PH domain containing protein	45641
143	L8H8H0	Formin domain containing protein	32305
144	L8HKE9	Myosin head (Motor domain) domain containing protein	156879
145	L8HG01	Proteasome subunit beta	23297
146	L8H6B5	Adenosylhomocysteinase	53078
147	L8GXW6	Profilin	13057
148	L8GRC8	Ras-related protein Rab-21, putative	13448
149	L8HMP8	Serine carboxypeptidase S28	59851
150	L8H5K0	Ammonium transporter subfamily protein	54529
151	L8GYM5	Ribosomal protein L15	23028
152	L8HDL4	Ras small GTP binding protein	21506
153	L8H9L6	Carboxylic ester hydrolase	57687
154	L8H1F0	TolAlike protein	35760
155	L8GI82	Peptidase family M13, putative	79879
157	L8HGQ4	Actin-related protein 2	44229
158	L8GYZ0	Ribosomal protein S8, putative	14776
159	L8HCV2	Actin binding protein	14412
160	L8GMD6	Calcium binding protein 39 (Mo25 protein) isoform 5, putative	37959
161	A0A060A630	M17 leucine aminopeptidase	61821

Protein group	Accession	Protein	Mass (Da)
162	L8GZ90	BAR domain containing protein	46092
163	L8H4M1	Uncharacterised protein	15613
164	L8GYD3	Epimerase domain-containing protein	40396
167	L8GHQ0	CMF receptor CMFR1, putative	51865
168	L8GZW9	AP-2 complex subunit alpha	115032
169	L8GGH2	EF hand domain containing protein	17262
170	L8HHY7	Ras-related protein Rab	23568
171	L8H1H6	Serine proteinase	38022
172	L8GYQ2	Ras-like GTP-binding protein YPT1, putative	23119
173	L8GPS7	Adaptorrelated protein complex 1, beta 1 subunit, isoform 2, putative	95802
175	L8GK29	Ras subfamily protein	28833
176	L8H7T7	Ras-like protein rasG, putative	21549
177	L8GZI4	Ctype lysozyme/alpha-lactalbumin superfamily protein	14743
178	L8GJ32	40S ribosomal protein S4	27273
179	L8HMC3	Actophorin, putative	15554
180	L8H5V6	LIM domain containing protein	15265
181	L8HCY6	EF hand domain containing protein	16054
182	A0A0S0ILC6	ATP synthase subunit 9, mitochondrial	8246
183	L8GZX0	Uncharacterised protein	17224
184	L8GW30	40S ribosomal protein S26	14634
185	L8HIU4	Deoxyribose-phosphate aldolase	42850
186	L8GLW9	Calmodulin, putative	16829
187	L8H6F0	ABC2 type transporter superfamily protein	78703
188	H9C893	Protein kinase C17	49527
189	L8HDH5	Dual specificity protein kinase	114598
190	L8GWQ5	Protein kinase domain containing protein	148656
191	L8GQ86	Sec1 family protein (Fragment)	50700
192	L8HIN1	AP complex subunit sigma	16618
193	L8H3X7	p21Rho-binding domain containing protein	45637
194	H9C877	Protein kinase C1	41292
195	L8HHU3	Ribosomal protein S15, putative	16941
197	L8HBF5	Rho-GAP domain containing protein	34491
198	L8GT08	Multifunctional fusion protein	62912
199	L8GVQ1	Vacuolar proton ATPase, putative	25615
200	L8HGE4	Uncharacterised protein	104847
201	L8GYW5	Vacuolar protein 8	90675
202	L8GG02	Uncharacterised protein (Fragment)	22813
206	L8GVI5	EF hand domain containing protein	24858
207	L8GF16	GTP-binding nuclear protein	24107
208	L8GTG8	Phospholipid-transporting ATPase	162640
209	L8HHV3	Syntaxin protein, putative	34388
210	L8HLC2	Guanine nucleotide-binding protein alpha-5 subunit, putative	40188
211	L8GLE7	Glycosyl hydrolase, putative	108173
212	L8GTE1	Isocitrate dehydrogenase [NADP]	45909
213	L8GVZ7	LBP / BPI / CETP family, Cterminal domain containing protein	50315
214	L8H2J7	Leucine rich repeat domain containing protein (Fragment)	26661
215	L8GG17	Proteasome subunit alpha type	27045
216	L8HIC7	Uncharacterised protein	31129

Protein group	Accession	Protein	Mass (Da)
217	L8H5X8	Uncharacterised protein	19931
218	L8GWC9	Uncharacterised protein	41242
219	L8HE70	Uncharacterised protein	25277
220	L8H3F2	Ras subfamily protein	58090
221	L8HET7	PH domain containing protein	52619
222	L8H7D8	Rho-GAP domain containing protein	23024
232	L8HEB0	DIL domain containing protein	122127
234	L8HDU2	Protein phosphatase 2C domain containing protein	97620
239	L8HK22	Serine/threonine-protein phosphatase	41035
240	L8HHZ7	Ras gtpase-activating family protein	22508
244	L8GTS0	Uncharacterised protein	22007
245	L8GKX4	Succinylglutamate desuccinylase/aspartoacylase, putative	58245
246	L8HMG4	Adenylyl cyclase-associated protein	51564
247	L8H089	NADP oxidoreductase coenzyme F420-dependent protein	66355
248	L8HH66	H(+)-transporting two-sector ATPase	64528
249	L8H5G5	20S proteasome alpha subunit F, putative	27658
250	L8GWU5	Zinc finger, C3HC4 type (RING finger) domain containing protein	63363
251	L8HDS9	Proteasome subunit alpha type	27947
252	L8GT12	Uncharacterised protein	14996
253	L8GRL5	Ribosomal protein L23, putative	23234
254	Q8WQZ8	Cysteine proteinase	36427
268	L8GMR5	Ras subfamily protein	21677
269	L8GXB4	Rab GDP dissociation inhibitor	45987
270	L8HKY5	Ribosomal protein S11, putative	8214
271	L8GKC5	Methyltransferase	39023
272	L8H6U2	Heat shock protein 90 alpha, putative	82639
273	L8H6Z7	RSNARE, VAMP71-family	22009
274	L8HK02	Serine/threonine-protein phosphatase	34985
275	L8H7G5	LIM domain containing protein	32017
276	L8HJ60	Mob4B protein isoform 3, putative	24767
277	L8GGS6	Rho-GAP domain containing protein	85401
278	L8GD46	Uncharacterised protein	35172
279	L8HL46	Ribosomal protein S17, putative	18302
280	L8H5Z9	Uncharacterised protein	28456
281	L8GQB4	Peptidase family m1 domain containing protein	137223
282	L8GID1	Universal stress domain containing protein	13112
283	L8GRK2	Mannose-P-dolichol utilisation defect 1 protein homolog	28199
284	L8GR44	Ras subfamily protein	19903
285	L8GN90	Histone H2A	14104
286	L8H869	Slime mold cyclic amp receptor protein	38157
287	L8GJ18	Dipeptidyl peptidase	84185
288	L8GJG4	Plastin 3 (T isoform), putative	57540
289	L8HM44	FYVE zinc finger domain/Ankyrin repeat containing protein	50355
290	L8GHL6	SCP family extracellular, putative	77922
291	L8GN71	HEAT repeat domain containing protein	178203
292	L8GWA3	Uncharacterised protein	31903
293	L8H4F3	Alkaline phosphatase	62306
294	L8GW17	Protein phosphatase 2C domain containing protein	62966

Protein group	Accession	Protein	Mass (Da)
295	L8H942	Ras-like protein 1, putative	18834
296	P90514	Glyceraldehyde-3-phophate dehydrogenase (Fragment)	5218
297	L8H1S4	Rho-GEF domain containing protein	88417
298	L8H358	UTP--glucose-1-phosphate uridylyltransferase	57000
299	L8H1X4	Ribosomal protein L3, putative	45111
300	L8H776	HTH cro/C1-type domain-containing protein	16658
301	L8H1A5	Rap/ran-GAP protein, putative	61553
302	L8HDQ1	Tetratricopeptide repeat domain containing protein	26834
303	L8GR77	Peptidyl-prolyl cis-trans isomerase	18521
304	L8HJ51	Aspartic proteinase (Fragment)	52867
305	L8GUG0	Leucine rich repeat domain containing protein	79620
306	L8GMC8	Uncharacterised protein	20184
307	L8HJ93	4-hydroxyphenylpyruvate dioxygenase	46216
308	A0A5H2WY43	Cysteine proteinase	36442

TABLE II

Identified proteins in extracellular vesicles (EVs) secreted by trophozoites of *Acanthamoeba* T5 incubated at 37°C

Protein group	Accession	Protein	Mass (Da)
1	L8HCZ7	Actin-1, putative	41676
2	L8GXI3	Rho family, small GTP binding protein Rac3, putative	21686
3	L8H9Q4	Ribosomal protein s27a, putative	17847
4	Q6WMU7	Ubiquitin-like protein Ublp94.4	94410
5	O61080	Myosin IC heavy chain	129461
6	O77202	Myosin-IA	134104
7	L8H1F4	Myosin VIIa, putative	232883
8	L8H6F1	p53 inducible protein	163453
9	L8H836	V-type proton ATPase proteolipid subunit	16580
10	P90630	Myosin-I binding protein Acan125	121610
11	L8HEA2	I/LWEQ domain containing protein	70416
12	L8HGB4	Rab-E family small GTPase (Fragment)	17380
13	L8GVM5	Talin, putative (Fragment)	120262
14	L8GRX4	Uncharacterised protein	21176
15	L8H373	14_3_3 domain-containing protein	36290
16	L8HKB9	Conditioned medium factor, putative	77482
17	L8H896	RAB1B, member RAS oncogene family	22332
18	L8GI20	Uncharacterised protein (Fragment)	18530
19	L8HD31	Ras-related protein ORAB-1, putative	23048
20	L8GG53	Longin domain-containing protein (Fragment)	21563
21	L8GJT1	Linoleate 8R-lipoxygenase	134070
22	H9C880	Protein kinase C4 (Fragment)	40357
23	L8HCM5	Calponin domain containing protein	67120
24	L8H4N4	Uncharacterised protein	22477
25	L8GR43	RAP1A, member of RAS oncogene family	22280
26	L8HHF3	Ras-related protein Rab-7A	20543
27	L8GZY0	Ras-related protein Rac1, putative	22515
29	L8H6G0	Guanylate cyclase	66526
31	L8H8P6	Ras-like protein 1, putative	22559
33	Q2TTE4	HSP70 (Fragment)	52239

Protein group	Accession	Protein	Mass (Da)
34	L8GZS5	Ras GTPase rap1b, putative	21717
35	L8GZK1	Elongation factor 1alpha, somatic form, putative (Fragment)	35896
36	L8GXE8	Ras subfamily protein, putative	33152
37	L8H806	Ras-related protein Rab-1A, putative	19134
38	L8HGS9	Rho guanine dissociation factor isoform 2, putative	22716
39	L8GHW5	C8 sterol isomerase	21546
40	L8HGF1	Ras subfamily protein	21544
41	L8HGZ3	Rab8/RabE family small GTPase, putative	22943
42	L8GSY8	Uncharacterised protein	131967
44	L8HAC7	Ras-related protein Rab	24602
45	L8GG56	High molecular weight heat shock protein	72288
46	L8HJ59	F-actin-capping protein subunit beta	31162
47	L8GT20	Eukaryotic translation elongation factor 2, putative	93342
48	L8GW48	PAKA subfamily protein kinase	56680
49	L8GIH3	Ras family GTPase	21879
50	L8GVH2	Phospholipid-transporting ATPase	129759
51	L8H015	Uncharacterised protein	234320
52	L8HJG5	Cell cycle control protein	36863
54	L8HD62	Acyloxyacyl hydrolase	66359
55	L8GUQ6	Uncharacterised protein	33579
56	L8HIM5	Acid stable alpha-amylase	53108
58	L8H263	Serine/threonine protein kinase	77930
59	L8H129	Fascin subfamily protein	14443
60	L8H7M2	Myosin1, putative	177415
61	L8HJ94	Rac-like protein (Fragment)	24794
62	L8H850	Ras-related protein Rap-1, putative	21517
63	L8HED5	C2 domain containing protein	14171
64	L8GNN8	LIM domain containing protein	18989
65	L8GHJ9	Gtpase-activator protein for Ras family gtpase	283475
66	L8GLH7	DIL domain containing protein	74709
67	L8HFB5	Glutamate dehydrogenase	51265
71	L8H946	Ras-related protein Rab-14	23259
72	L8GF41	14_3_3 domain-containing protein	29440
73	L8GGY5	ABC2 type transporter superfamily protein	169171
74	L8GXA8	Copine VIII, putative	64292
75	L8HDD8	Guanylate cyclase	175931
77	L8H4B9	SH3 domain containing protein	40786
79	L8GW76	RAC GTPase	18654
80	L8HM01	Uncharacterised protein	57527
83	L8HKE9	Myosin head (Motor domain) domain containing protein	156879
85	L8GLB7	Purinergic receptor, putative	39667
86	L8H6R4	Peroxidase ppod1, putative	14178
87	L8HG55	Ras subfamily protein	18012
88	L8HKD4	Uncharacterised protein	64553
90	L8GY18	Protein kinase domain containing protein	111296
91	L8GQ45	Peptidyl-prolyl cis-trans isomerase	18101
92	L8GTG8	Phospholipid-transporting ATPase	162640
93	L8GWZ2	Arp2/3 complex 34 kDa subunit	33355
94	L8GG08	Actin bundling protein	31046
95	L8H8C6	Protein kinase domain containing protein	52031

Protein group	Accession	Protein	Mass (Da)
96	L8H8H0	Formin domain containing protein	32305
97	L8H1E5	F-actin-capping protein subunit alpha	30721
98	L8HE57	C2 domain containing protein	14182
99	L8GFZ9	Coronin	50119
100	L8GQE7	Vacuolar protein 8	54138
101	H9C898	Protein kinase C22	45499
102	L8H599	Ras-related protein ralB-B, putative	20317
103	L8GUU1	Serine/threonine kinase	39217
104	L8HDK5	Actin-related protein 3, putative	47983
105	B0FYM3	Serine proteinase	43788
106	L8HJ43	RAB11B protein, putative	23438
107	Q6QU59	Coronin	50143
108	L8HE18	Rho family, small GTP binding protein Rac3, putative	21431
109	L8H9U4	ABC2 type transporter superfamily protein	76091
110	L8HKL1	PH domain containing protein	45641
111	L8HCU4	Guanine nucleotide binding protein G(O), alpha subunit, putative	40681
112	L8HMC3	Actophorin, putative	15554
113	L8HJ50	Uncharacterised protein	22572
114	L8H0P3	40S ribosomal protein S12	16613
115	L8GI71	S-adenosylmethionine synthase	42531
116	L8GUS9	Ras-like protein	27427
117	L8HBH8	Non-specific serine/threonine protein kinase	47151
118	L8GGA4	Myosin IF, putative	111359
119	H9C877	Protein kinase C1	41292
120	H9C893	Protein kinase C17	49527
122	L8H5T5	Guanylate cyclase	168120
123	L8HHZ1	V-type proton ATPase subunit a	92623
124	L8H9P5	DOCK family protein	190306
125	L8GYD3	Epimerase domain-containing protein	40396
126	L8H6I9	SH3 domain containing protein	56923
127	L8HA95	Rho-GEF domain containing protein	96754
128	L8GI82	Peptidase family M13, putative	79879
129	L8H122	Vacuolar proton pump d subunit, putative	35205
130	L8GD76	Terpene cyclase/mutase family member	81940
131	L8GTS0	Uncharacterised protein	22007
132	L8HMP8	Serine carboxypeptidase S28	59851
133	L8H6U2	Heat shock protein 90 alpha, putative	82639
134	L8H192	Actin-related protein 2/3 complex subunit 5	13950
135	L8H398	Toll-like protein	34082
136	L8H1H5	F_actin_bund_C domain-containing protein	37657
138	L8HDL4	Ras small GTPbinding protein	21506
139	L8H7T7	Ras-like protein rasG, putative	21549
140	L8GTN6	Catalase	55347
141	L8GQT3	40S ribosomal protein S3a	29244
142	L8H8F6	Phosphoesterase family protein	57246
143	L8GVG0	Toll-like protein	33223
144	L8GVF2	Ras family protein	22331
145	L8GZW9	AP-2 complex subunit alpha	115032
146	L8HH66	H(+)-transporting two-sector ATPase	64528
147	B0FYM4	Encystation-mediating serine proteinase	42797

Protein group	Accession	Protein	Mass (Da)
148	L8H942	Ras-like protein 1, putative	18834
149	L8HGQ4	Actin-related protein 2	44229
150	L8HE70	Uncharacterised protein	25277
151	L8GPZ0	Laminin egf-like (Domains iii and v) domain containing protein (Fragment)	193141
152	L8GMD6	Calcium binding protein 39 (Mo25 protein) isoform 5, putative	37959
153	L8GHQ0	CMF receptor CMFR1, putative	51865
154	L8HE67	Ankyrin repeat containing protein	16126
155	L8GXW6	Profilin	13057
156	L8GF83	Peroxiredoxin 2, putative	22119
157	L8GSP6	Ras-related protein Rab-2A, putative	22614
158	L8HBF6	Profilin	13084
159	L8GFL5	Peroxidase	23233
160	L8GK29	Ras subfamily protein	28833
161	L8H082	Communication mutant protein f, putative	116632
162	L8GMR5	Ras subfamily protein	21677
163	L8H5K0	Ammonium transporter subfamily protein	54529
164	L8HDH5	Dual specificity protein kinase	114598
165	L8HC07	ABC2 type transporter superfamily protein	158067
166	L8GTD0	Chorismate mutase subfamily protein	20759
167	L8H5M1	Peptidase M20, putative	48755
170	L8GPS7	Adaptorrelated protein complex 1, beta 1 subunit, isoform 2, putative	95802
171	L8H9L6	Carboxylic ester hydrolase	57687
172	L8HBF5	Rho-GAP domain containing protein	34491
173	L8H1H6	Serine proteinase	38022
175	L8GX28	Rho-GAP domain containing protein	116213
176	L8HCV2	Actin binding protein	14412
177	L8GLW9	Calmodulin, putative	16829
180	L8GTE1	Isocitrate dehydrogenase [NADP]	45909
181	L8GLE7	Glycosyl hydrolase, putative	108173
182	L8GM46	Uncharacterised protein	66861
183	L8GGH2	EF hand domain containing protein	17262
184	L8HHY7	Ras-related protein Rab	23568
185	L8GVI5	EF hand domain containing protein	24858
186	L8GG02	Uncharacterised protein (Fragment)	22813
187	L8H6B5	Adenosylhomocysteinase	53078
189	L8GYQ2	Ras-like GTP-binding protein YPT1, putative	23119
190	L8GZ90	BAR domain containing protein	46092
191	L8GXB4	Rab GDP dissociation inhibitor	45987
192	L8GQE5	Nucleoside diphosphate kinase	25432
193	L8GVQ1	Vacuolar proton ATPase, putative	25615
194	L8GMR7	Eukaryotic porin protein	33586
195	L8H5X8	Uncharacterised protein	19931
196	L8H5V6	LIM domain containing protein	15265
197	L8GRC8	Ras-related protein Rab-21, putative	13448
198	L8H3F2	Ras subfamily protein	58090
199	L8HIU4	Deoxyribose-phosphate aldolase	42850
200	L8H4F9	Phosphatidylinositol-3-phosphatase (Fragment)	113154
201	L8GLU0	Ribosomal protein S4, putative (Fragment)	21839
202	L8HLC2	Guanine nucleotide-binding protein alpha-5 subunit, putative	40188
203	L8GH69	Beta-N-acetylhexosaminidase (Fragment)	39794

Protein group	Accession	Protein	Mass (Da)
204	L8HHZ7	Ras gtpase-activating family protein	22508
205	L8GNH8	60S ribosomal protein L7a	31732
206	L8HMG4	Adenylyl cyclase-associated protein	51564
207	L8GZI4	Ctype lysozyme/alpha-lactalbumin superfamily protein	14743
208	L8H790	PH domain/RhoGEF domain containing protein	98158
209	L8H2Q1	Elongation factor 1-gamma family protein	46387
210	L8GYM5	Ribosomal protein L15	23028
211	L8GWU5	Zinc finger, C3HC4 type (RING finger) domain containing protein	63363
212	L8HDU2	Protein phosphatase 2C domain containing protein	97620
213	L8GMG4	Endonuclease	33808
214	L8HCY6	EF hand domain containing protein	16054
215	L8H3X7	p21Rho-binding domain containing protein	45637
217	L8GWC9	Uncharacterised protein	41242
218	L8GG28	Mitogen-activated protein kinase	47967
219	L8HG01	Proteasome subunit beta	23297
220	L8GF16	GTP-binding nuclear protein	24107
221	A0A060A630	M17 leucine aminopeptidase	61821
222	L8GG17	Proteasome subunit alpha type	27045
223	L8H2J7	Leucine rich repeat domain containing protein (Fragment)	26661
224	L8H5G5	20S proteasome alpha subunit F, putative	27658
225	L8GYZ0	Ribosomal protein S8, putative	14776
226	L8H6Z7	RSNARE, VAMP71-family	22009
227	L8H869	Slime mold cyclic amp receptor protein	38157
228	L8H5W6	Calpain large subunit, domain iii domain containing protein	65973
229	L8GRK2	Mannose-P-dolichol utilisation defect 1 protein homolog	28199
230	L8HIC7	Uncharacterised protein	31129
231	L8H4M1	Uncharacterised protein	15613
232	L8GYW5	Vacuolar protein 8	90675
233	A0A0K1HQ82	ATP synthase subunit 9, mitochondrial	8246
234	L8HGE4	Uncharacterised protein	104847
235	L8H776	HTH cro/C1-type domain-containing protein	16658
236	P90514	Glyceraldehyde-3-phophate dehydrogenase (Fragment)	5218
237	L8GWQ5	Protein kinase domain containing protein	148656
238	L8GK33	Amine oxidase	76094
244	L8HEB0	DIL domain containing protein	122127
247	L8HM44	FYVE zinc finger domain/Ankyrin repeatcontaining protein	50355
248	L8GRZ9	Phosphopyruvate hydratase	46589
249	L8HAN3	3phosphoinositide-dependent protein kinase 1, putative	60719
251	L8GQ86	Sec1 family protein (Fragment)	50700
252	L8GG04	Vacuolar protein sorting-associated protein 29	69133
255	L8H5Z9	Uncharacterised protein	28456
256	L8HHV3	Syntaxin protein, putative	34388
258	L8GN77	Protein kinase	40242
265	L8GI34	LIM domain containing protein	39651
266	L8H4P8	Guanine nucleotide-binding protein beta subunit, putative	35215
267	L8H7D8	Rho-GAP domain containing protein	23024
268	L8HGI4	Uncharacterised protein	117771
269	L8GFD3	Uncharacterised protein	23502
270	L8H089	NADP oxidoreductase coenzyme F420-dependent protein	66355
271	L8GVZ7	LBP / BPI / CETP family, Cterminal domain containing protein	50315

Protein group	Accession	Protein	Mass (Da)
272	L8H5Z5	Dipeptidyl-peptidase family protein	54723
273	L8HKF5	Uncharacterised protein (Fragment)	25920
275	L8GJ32	40S ribosomal protein S4	27273
276	L8HH94	Protein kinase domain containing protein	92617
277	Q8WQZ8	Cysteine proteinase	36427
278	L8HBE7	Villin headpiece domain containing protein	111071
279	L8GJY4	ABC transporter, ATPbinding domain containing protein	69338
280	A0A5H2WY43	Cysteine proteinase	36442
281	L8GV82	Rho-GAP domain containing protein	78146
285	L8GY40	Ubiquitin B isoform 2, putative	12653
295	L8HBA1	Beta_helix domain-containing protein	52920
296	L8GKC5	Methyltransferase	39023
297	L8HJ51	Aspartic proteinase (Fragment)	52867
298	L8GTU9	Uncharacterised protein	32116
299	L8HK02	Serine/threonine-protein phosphatase	34985
300	L8H533	Calponin domain containing protein	15129
301	L8GLP8	Ribosomal protein L35Ae, putative	12290
302	L8GD46	Uncharacterised protein	35172
303	L8HCE4	IRSp53/MIM domain containing protein	39784
304	L8HL59	Leucine rich repeat protein lrrA, putative	56741
305	L8GR44	Ras subfamily protein	19903
306	L8H880	Glycosyl hydrolases family 25 subfamily protein	70775
307	L8GJG4	Plastin 3 (T isoform), putative	57540
308	H9C888	Protein kinase C12	57429
309	L8HL46	Ribosomal protein S17, putative	18302
310	L8GJ18	Dipeptidyl peptidase	84185
311	L8HC03	Transport protein Sec61 alpha subunit, putative	51053
312	L8H8T3	Domain found in dishevelled, egl10, and pleckstrin domain containing protein	74645
313	L8HKI3	Glycogen synthase kinase 3 alpha, putative	46415
314	L8GN90	Histone H2A	14104
315	A0A1D8D6M0	Cytochrome-c oxidase	99214
316	L8HET7	PH domain containing protein	52619
317	L8HK93	Uncharacterised protein	27624
318	L8H4F3	Alkaline phosphatase	62306
319	L8HAJ0	AcylCoA dehydrogenase, C-terminal domain containing protein	69295
320	L8HGV0	Proteasome subunit alpha type	27116
321	L8GN55	Kelch repeat containing protein	33956
322	L8HIW2	Uncharacterised protein	81810
323	L8GZ46	PH domain containing protein	23392
324	L8GZX0	Uncharacterised protein	17224

TABLE III

Identified proteins in ACM secreted by trophozoites of *Acanthamoeba* T5 incubated at 28°C (band of ~140 bp)

Protein group	Accession	Protein	Mass (Da)
1	L8HKE1	Xylose isomerase	49529
2	L8GX42	Zinc carboxypeptidase superfamily protein	48145
3	L8GJ18	Dipeptidyl peptidase	84185
4	L8H3J6	Alcohol dehydrogenase	41290
5	Q5IZD9	Superoxide dismutase	22593
6	L8GUI9	Uncharacterised protein	19167
7	L8H880	Glycosyl hydrolases family 25 subfamily protein	70775
8	L8HLJ4	Carboxypeptidase A3, putative	46979
9	B0FYM3	Serine proteinase	43788
10	L8GZM6	GPI anchored protein	54871
11	L8GSI3	FAD binding domain containing protein	52342
12	L8GI71	S-adenosylmethionine synthase	42531
13	L8GUQ6	Uncharacterised protein	33579

TABLE IV

Identified proteins in ACM secreted by trophozoites of *Acanthamoeba* T5 incubated at 28°C (band of ~260 bp)

Protein group	Accession	Protein	Mass (Da)
1	L8GX61	Alpha-mannosidase	111557
2	L8HGR3	Amidohydrolase domain containing protein	92127
3	L8HD44	Glucuronidase, beta, putative	45236
4	L8GUL5	Glycosyl hydrolase (Fragment)	48406
5	L8GXZ7	Xylosidase	89226
6	L8GJS6	Inosineuridine preferring nucleoside hydrolase family protein	40838
7	L8GM39	Aspartyl aminopeptidase	40797
8	L8GU40	Uncharacterised protein	71449
9	L8GPK5	Aspartyl aminopeptidase	15170
10	L8GNH0	4aminobutyrate aminotransferase	48458
11	L8GXW7	Mannose binding protein	38694
12	L8HKQ5	Amidohydrolase domain containing protein	123298
13	L8GX42	Zinc carboxypeptidase superfamily protein	48145
14	L8HEN9	Mannosidase, beta A, lysosomal, putative	19723
15	L8H821	Aldehyde oxidase and xanthine dehydrogenase, molybdopterin binding domain containing protein	146240
16	A0A060A630	M17 leucine aminopeptidase	61821
18	Q5IZD9	Superoxide dismutase	22593
19	L8HF66	Rho-GAP domain containing protein	57616
20	L8GUQ6	Uncharacterised protein	33579
21	L8H880	Glycosyl hydrolases family 25 subfamily protein	70775
22	L8HLJ4	Carboxypeptidase A3, putative	46979
23	L8GUI9	Uncharacterised protein	19167
25	L8GZM6	GPI anchored protein	54871
29	L8H0Z8	Thiazole biosynthesis protein ThiG, putative	29867
30	L8HAL7	Glycosyl hydrolase domain containing protein	61519

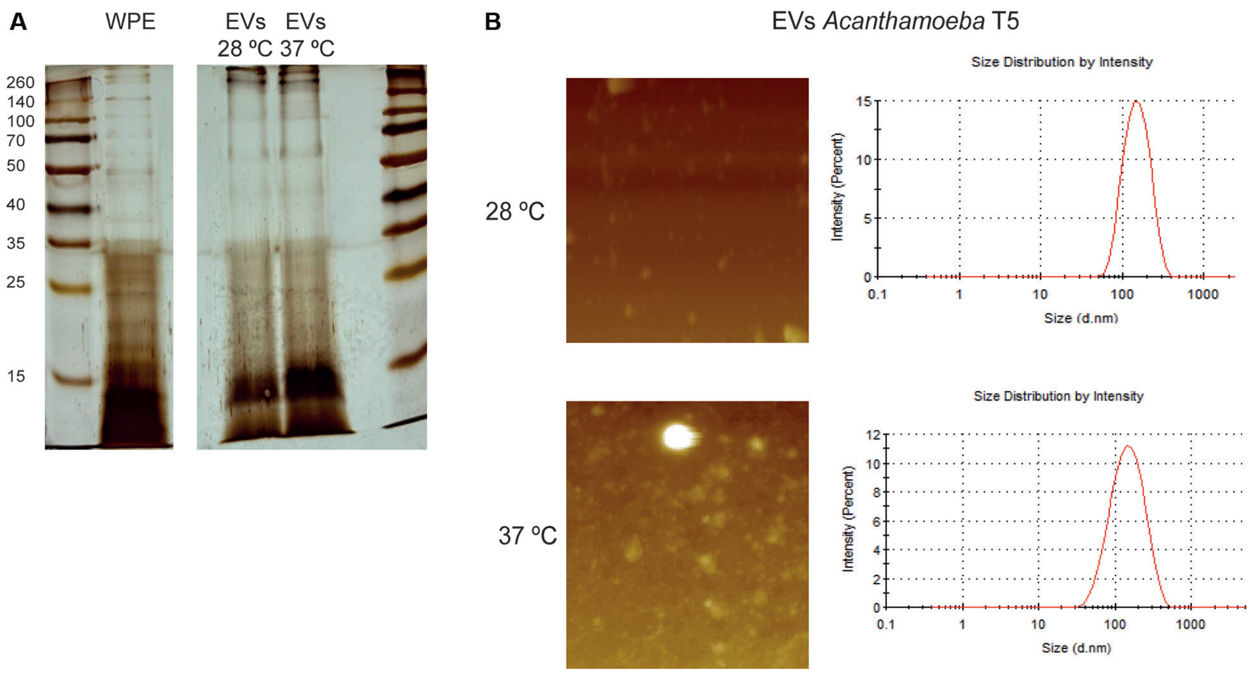

Fig. 1: isolation of extracellular vesicles by trophozoites of *Acanthamoeba* T5 incubated at 28ºC and 37ºC: (A) protein profile visualised after silver staining; (B) atomic force microscopy and dynamic light scattering analyses that revealed mean values of 128.7 ± 52.77 nm for extracellular vesicles (EVs) released at 28ºC and 128.2 ± 61.54 for EVs released at 37ºC. WPE: whole protein extracts of a lysate of trophozoites of *Acanthamoeba* T5.

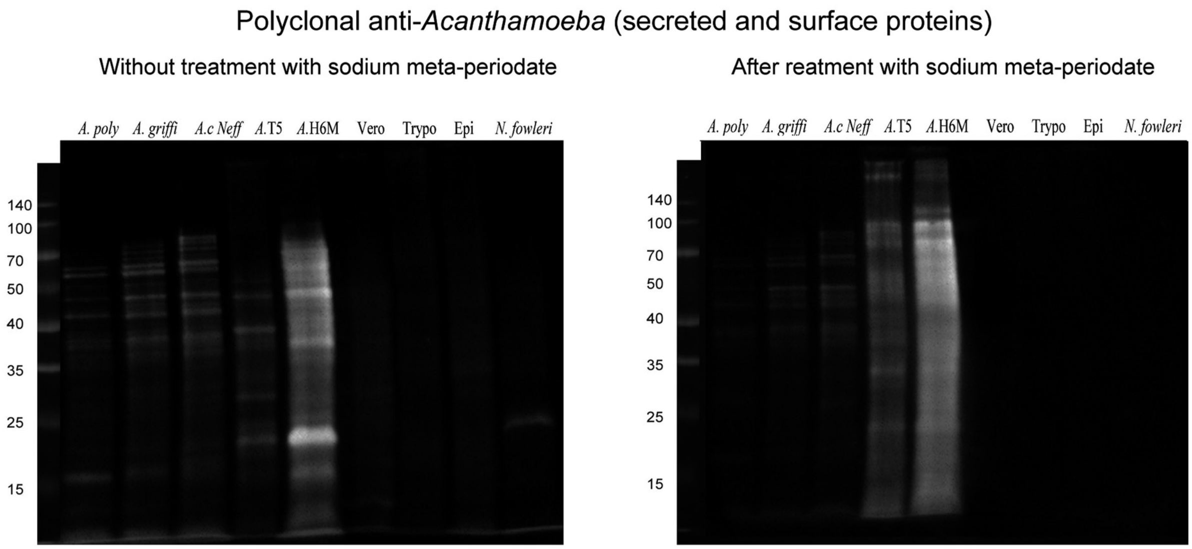

Fig. 2: confirmation of the presence of non-glycosilated proteins in lysates of trophozoites of *Acanthamoeba* after the incubation of the Western blot membrane with 10 mg/mL sodium *meta*-periodate, prior to the challenge with the antibodies produced with secreted and surface proteins.
